# Rutin Exerts Cytotoxic and Senescence-Inducing Properties in Human Melanoma Cells

**DOI:** 10.3390/toxics9090226

**Published:** 2021-09-19

**Authors:** Iulia Pinzaru, Raul Chioibas, Iasmina Marcovici, Dorina Coricovac, Razvan Susan, Denisa Predut, Doina Georgescu, Cristina Dehelean

**Affiliations:** 1 Research Center for Pharmaco-Toxicological Evaluations, Faculty of Pharmacy, “Victor Babeș” University of Medicine and Pharmacy Timisoara, Eftimie Murgu Square No. 2, 300041 Timisoara, Romania; iuliapinzaru@umft.ro (I.P.); iasmina.marcovici@umft.ro (I.M.); cadehelean@umft.ro (C.D.); 2 Faculty of Pharmacy, “Victor Babeș” University of Medicine and Pharmacy Timisoara, Eftimie Murgu Square No. 2, 300041 Timisoara, Romania; 3 Faculty of Medicine, “Victor Babeș” University of Medicine and Pharmacy Timisoara, Eftimie Murgu Square No. 2, 300041 Timisoara, Romania; office@medcom.ro (R.C.); georgescu.doina@umft.ro (D.G.); 4 Faculty of Dental Medicine, “Victor Babeș” University of Medicine and Pharmacy Timisoara, Eftimie Murgu Square No. 2, 300041 Timisoara, Romania; antoaneta.predut@farmaciiledona.ro

**Keywords:** rutin, human melanoma cells, cell viability, cellular senescence

## Abstract

Malignant melanoma represents the deadliest type of skin cancer with narrow treatment options in advanced stages. Herbal constituents possessing anticancer properties occupy a particular spot in melanoma research as potential chemotherapeutics. Rutin (RUT) is a natural compound exerting antioxidant, antimicrobial, anti-inflammatory, UV-filtering, and SPF-enhancing activities that are beneficial to the skin; however, its effect as an anti-melanoma agent is less investigated. The current study is focused on assessing the cytotoxic potential of RUT against two different human melanoma cell lines: RPMI-7951 and SK-MEL-28 by evaluating its impact in terms of cell viability, cells’ morphology, and nuclear aspect assessment, and senescence-inducing properties. The results indicate a dose-dependent decrease in the viability of both cell lines, with calculated IC_50_ values of 64.49 ± 13.27 µM for RPMI-7951 cells and 47.44 ± 2.41 µM for SK-MEL-28, respectively, accompanied by a visible reduction in the cell confluency and apoptotic features within the cell nuclei. RUT exerted a senescence-inducing property highlighted by the elevated expression of senescent-associated beta-galactosidase (SA-β-gal) in SK-MEL-28 cells. Despite the in vitro anti-melanoma effect revealed by our results, further studies are required to elucidate the mechanisms of RUT-induced cytotoxicity and senescence in melanoma cells.

## 1. Introduction

Skin serves as the main physical, chemical, and immunological barrier against environmental noxious agents [1,2], as well as one of the most common routes of toxics’ entrance into the body [3]. Therefore, it is the most vulnerable target to external damage [4], which occurs when the abnormal exposure to external stressors such as ozone (O_3_), air pollutants, and ultraviolet (UV) light surpasses the skin’s protective capacity [3,5]. The major mechanism underlying the detrimental effect of environmental insults on the skin is through the generation of oxidative stress which depletes the cutaneous antioxidant capacity [3]. The alteration of skin barrier function leads to the development of various cutaneous diseases, including erythema, edema, dermatitis, psoriasis, photoaging, and cancer [3].

Carcinogenesis remains the most severe consequence of the prolonged exposure of the skin to environmental carcinogens. The incidence of skin cancers, both melanoma, and non-melanoma subtypes, is continuously rising [6]. Non-melanoma skin cancers (i.e., basal cell and squamous cell carcinomas) are among the most frequent cancer types and are generally treated by surgical excision [7]. On the other hand, despite its rareness [8], melanoma—the tumor arising from the malignant transformation of melanocytes [9], remains the leading cause of skin cancer-related deaths worldwide [10]. Several effective drugs are currently available for melanoma treatment [11], but due to the severe side effects and tumor resistance to therapy, the success rates remain low [12,13]. In this context, the discovery and development of improved treatment strategies are indispensable and demand continuous research. Herbal constituents are widely embraced as complementary or alternative options in the field of oncology as chemopreventive agents, chemotherapeutics, or sensitizers [14]. In particular, natural compounds generally serve as potent anti-melanoma agents by initiating apoptosis and inhibiting cancer cell proliferation and metastasis [12].

Rutin (RUT) is a flavonol glycoside found in numerous plants (e.g., *Ruta graveolens* L., *Sophora japonica* L.) and food beverages such as citrus, apples, vegetables, buckwheat, black tea, etc. [15]. Due to its broad spectrum of pharmacological activities (e.g., antioxidant and ROS-scavenging properties; antibacterial activity; anti-inflammatory and antimutagenic effects), this small molecule has been brought into the spotlight of modern research aimed to assess its potential as a therapeutic agent in various pharmaceutical formulations or cosmeceuticals [16,17,18]. Several studies indicate the efficacy of RUT in counteracting skin injuries induced by environmental stressors [19,20,21,22]. For instance, RUT proved to be active against UV radiation-induced damage due to its structural similarity to organic UV filters and its strong antioxidant activity [19]. Additionally, RUT reduced skin photoaging by strengthening the cutaneous density and elasticity through the regulation of extracellular matrix enzymes [20], provided a sun protective factor (SPF) enhancement [21], and inhibited the UVB-induced inflammatory responses (i.e., cyclooxygenase-2 and inducible nitric oxide synthase expressions) [20]. The antitumor activity of RUT has been also demonstrated against various cancers (e.g., breast, colon, liver, lung) by targeting a multitude of apoptotic (Bcl-2, Bcl-2 associated X protein, caspases, Bax), autophagic (Beclin1, Atg5/12, LC3-II), inflammatory (nuclear factor-κB, interleukins), and angiogenic (vascular endothelial growth factor) signaling mediators [22]. However, the anticancer activity of RUT against skin carcinomas and its underlying molecular mechanisms have not been extensively investigated so far. Our recent publications offer preliminary insight into the pro-apoptotic effect exerted by RUT and its inclusion complexes in vitro in murine (B164A5) and human (A375) melanoma cells [15,23], still, the anticancer effect of rutin in melanoma is far from being elucidated.

The present study aims at providing an in vitro insight into the cytotoxic potential of RUT against two different human melanoma cell lines: RPMI-7951 (epithelial morphology) and SK-MEL-28 (polygonal morphology) by evaluating its impact in terms of cell viability, cells’ morphology, and nuclear aspect assessment, and senescence-inducing properties.

## 2. Materials and Methods

### 2.1. Reagents

Rutin, phosphate saline buffer (PBS), trypsin-EDTA solution, dimethyl sulfoxide (DMSO), fetal bovine serum (FBS), penicillin/streptomycin, and MTT reagent were purchased from Sigma Aldrich, Merck KgaA (Darmstadt, DE). The cell culture medium, Eagle’s Minimum Essential Medium (EMEM-ATCC^®^ 30-2003™), was acquired from ATCC (American Type Cell Collection, Lomianki, PL). The Senescence Detection Kit (ab65351) was purchased from Abcam (Cambridge, UK). All reagents were of analytical grade of purity and suitable for cell culture use.

### 2.2. Cell Culture

The experiments were performed on two human malignant melanoma cell lines provided from ATCC as frozen vials—RPMI-7951 (HTB-66™) and SK-MEL-28 (HTB-72™). The cell lines were cultured in their specific growth medium (EMEM) according to the manufacturer’s recommendations and were supplemented with 10% FCS and 1% antibiotics solution (100 U/mL penicillin/ 100 µg/mL streptomycin). The cells were incubated in a humidified atmosphere at 37 °C and 5% CO_2_.

### 2.3. Cell Viability Assessment

The MTT (3-(4,5-dimethylthiazol-2-yl)-2,5-diphenyltetrazolium bromide) assay was applied to evaluate RUT’s impact on melanoma cells viability. Thus, RPMI-7951 and SK-MEL-28 cells were seeded in 96-well plates (10^4^ cells/200 µL/well) and treated with increasing concentrations (1, 5, 10, 25, and 50 µM) of RUT for 24 h. The solvent used to prepare the stock solution of RUT was DMSO. At the end of the 24 h treatment, the old culture media was replaced with 100 µL of fresh media and 10 µL of MTT reagent/well, followed by a 3 h incubation at 37 °C. Finally, the solubilization solution (100 µL/well) was added, the plates were maintained protected from light, at room temperature for 30 min, and the absorbances values were measured at two wavelengths (570 and 630 nm) using Cytation 5 (BioTek Instruments Inc., Winooski, VT, USA).

### 2.4. Cell Morphology and Confluence Evaluation

To verify the impact of RUT on the morphology and confluence of RPMI-7951 and SK-MEL-28 cells, a microscopic examination was performed. The cells were observed under bright field illumination and photographed at the end of the 24 h treatment period using Cytation 1 (BioTek Instruments Inc., Winooski, VT, USA). The photographs were processed using the Gen5™ Microplate Data Collection and Analysis Software (BioTek Instruments Inc., Winooski, VT, USA).

### 2.5. Nuclear Morphology Assessment

The potential toxicity of RUT at the nuclear level was tested by applying the Hoechst 33342 staining assay protocol according to the manufacturer’s (Thermo Fisher Scientific, Inc., Waltham, MA, USA) recommendations. In brief, the RPMI-7951 and SK-MEL-28 cells were seeded in 12-well plates (10^5^ cells/1.5 mL/well) and treated with three selected concentrations (1, 10, and 50 µM) of RUT in DMSO for 24 h. After the stimulation period, the media was removed, and the staining solution diluted at 1:2000 in PBS was added (500 µL/well). The plates were incubated for 10 min at room temperature, protected from light. Finally, the staining solution was washed with PBS and the pictures were taken using Cytation 1 (BioTek Instruments Inc., Winooski, VT, USA) and analyzed by the means of Gen5™ Microplate Data Collection and Analysis Software (BioTek Instruments Inc., Winooski, VT, USA). Staurosporine (STP) 5 µM was selected as a positive control for apoptosis. The apoptotic index (AI) was calculated according to a formula described by Xu et al. [24]:

$$\mathrm{AI}=\frac{Number\;of\;apoptotic\;cells}{Total\;number\;of\;cells}\;\times \;100$$

### 2.6. Senescence Detection

To evaluate the influence of RUT (1, 10, and 50 µM) on the senescence process within melanoma cells, the expression of senescence-associated β-galactosidase (SA-β-Gal) was detected at the end of a 24 h treatment by performing the manufacturer’s protocol. In brief, for this experiment SK-MEL-28 cells were seeded in 12-well plates and treated for 24 h with RUT. At the end of the treatment, the cells were washed with PBS, fixed with the provided Fixing Solution, and stained using the provided Staining Solution which was supplemented with an X-gal solution in DMSO and a Staining Supplement. Finally, the cells were photographed in Color Bright Field illumination using the Olympus IX73 inverted microscope (Olympus, Tokyo, Japan).

### 2.7. Statistical Analysis

The data obtained were presented as means ± SD, and the differences were compared by one-way ANOVA, followed by Dunnett’s multiple comparison post hoc test by using GraphPad Prism version 6.0.0 for Windows (GraphPad Software, San Diego, CA, USA, www.graphpad.com). The differences between data were considered statistically significant if *p* < 0.1 and are labeled with * (* *p* <0.1, ** *p* < 0.01, *** *p* < 0.001 and **** *p* < 0.0001). 

## 3. Results

### 3.1. Cell Viability Assessment

**Figure 1:**
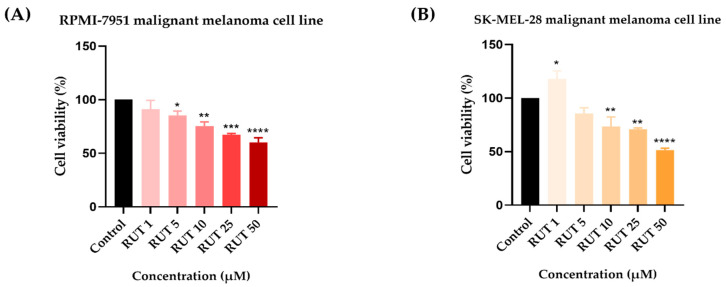
In vitro evaluation of the effect exerted by RUT (1, 5, 10, 25, and 50 µM) after 24 h of treatment on (**A**) RPMI-7951 and (**B**) SK-MEL-28 malignant melanoma cells’ viability by performing the MTT assay. Data are presented as viability percentages (%) normalized to Control and expressed as mean values ± SD of three independent experiments performed in triplicate. The statistical differences between Control and the treated group were verified by applying the one-way ANOVA analysis followed by Dunnett’s multiple comparisons post-test (* *p* < 0.1; ** *p* < 0.01; *** *p* < 0.001; **** *p* < 0.0001).

To identify the potential in vitro cytotoxic activity of RUT against melanoma cells, as well as its active concentrations, a cell viability assessment was performed. The 24 h treatment with RUT (1, 5, 10, 25, and 50 µM) indicated a dose-dependent decrease in the percentage of viable cells in both cell lines tested as compared to Control (Figure 1). In RPMI-7951 cells, the viability was significantly reduced even at low concentrations (5 µM–85.31%). However, the most prominent effect has been noticed at the highest concentration tested (50 µM–60%). In SK-MEL-28 cells, a significant increase in the cell viability was noticed at the lowest concentration (1 µM–117.84%), followed by a considerable reduction at higher concentrations up to 51.48% (at 50 µM). By comparing the calculated IC_50_ values of RUT in the two melanoma cell lines it has been remarked that SK-MEL-28 (IC_50_ = 47.44 ± 2.41) is more sensitive as compared to RPMI-7951 (IC_50_ = 64.49 ± 13.27).

### 3.2. Cell Morphology and Confluence Evaluation

**Figure 2:**
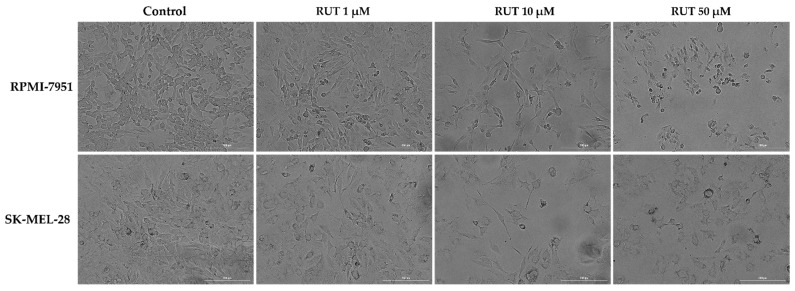
Morphological aspect and the confluence of RPMI-7951 and SK-MEL-28 melanoma cells following the 24 h treatment with RUT 1, 10, and 50 µM. The scale bars represent 200 µm.

As a component of the anti-melanoma profile of RUT, an evaluation of its impact on the morphology of RPMI-7951 and SK-MEL-28 cells has been performed. RPMI-7951 cells are adherent cells with an epithelial-like morphology and several changes were identified in their morphology and confluence following RUT treatment (Figure 2), such as a dose-dependent reduction of confluence, loss of adherence, and roundish cells were observed mainly at 10 and 50 µM. A similar effect was detected in the case of SK-MEL-28 cells, human melanoma cells with a polygonal morphology, the most noticeable signs being recorded at the highest concentrations tested—10 and 50 µM (Figure 2). The changes observed in both human melanoma cells indicate the cytotoxic effect of RUT treatment and could be characterized as apoptotic-specific signs. These data support the cell viability results. 

### 3.3. Nuclear Morphology Evaluation

**Figure 3:**
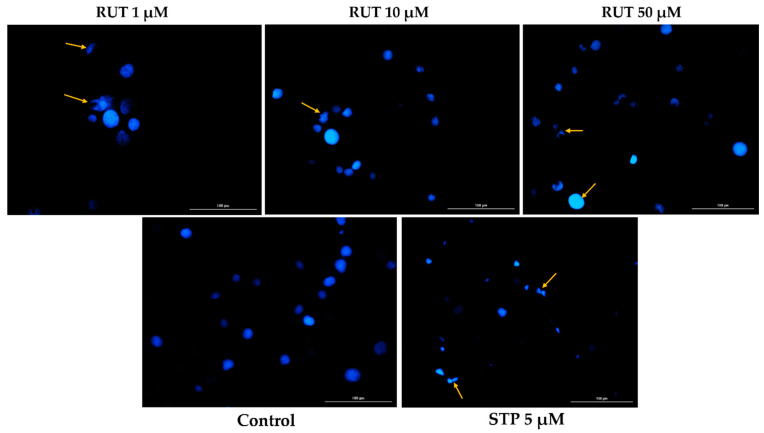
Hoechst 33342 staining of RPMI-7951 cells’ nuclei following the 24 h treatment with RUT 1, 10, and 50 µM. The yellow arrows indicate nuclei expressing abnormal features. The scale bars represent 100 µm.

**Figure 4:**
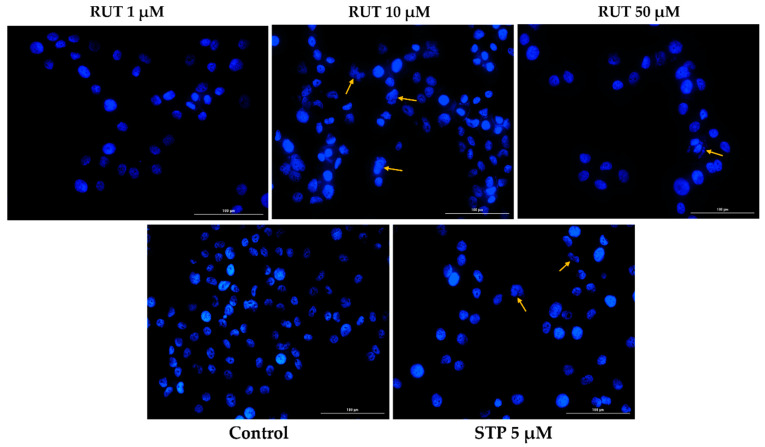
Hoechst 33342 staining of SK-MEL-28 cells’ nuclei following the 24 h treatment with RUT 1, 10, and 50 µM. The yellow arrows indicate nuclei expressing abnormal features. The scale bars represent 100 µm.

**Figure 5:**
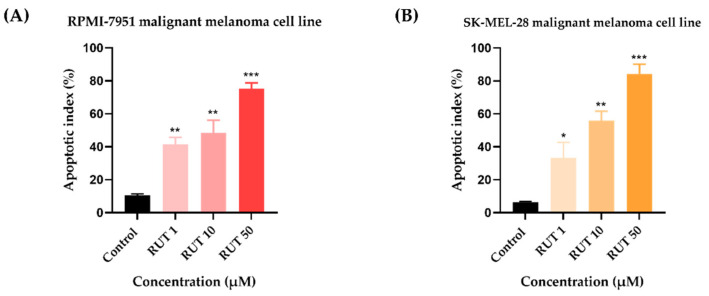
Calculated apoptotic index (AI) in Hoechst 33342 stained RPMI-7951 (**A**) and SK-MEL-28 (**B**) MM cells following the 24 h treatment with RUT (1, 10, and 50 µM). Data are presented as an apoptotic index (%) normalized to Control and expressed as mean values ± SD of three independent experiments. The statistical differences between Control and the treated group were verified by applying the one-way ANOVA analysis followed by Dunnett’s multiple comparisons post-test (* *p* < 0.1; ** *p* < 0.01; *** *p* < 0.001).

Since specific changes in the morphology of cells nuclei offer insight into the possible cell death mechanism induced by anticancer compounds, a Hoechst 33,342 staining was conducted for RUT 1, 10, and 50 µM. Staurosporine (STP) 5 µM was selected as an indicator for apoptosis. The interpretation of the pictures was performed according to Crowley and colleagues [25]. Several apoptotic features were noticed. In RPMI-7951 cells (Figure 3), RUT induced nuclear fragmentation (at 1 µM), membrane blebbing (at 10 µM), and chromatin condensation (at 50 µM). Abnormally shaped and condensed nuclei can be remarked at 10 µM in SK-MEL-28 cells (Figure 4), as well as nuclear fragmentation and apoptotic bodies at 50 µM. Additionally, the Hoechst staining revealed a dose-dependent increase in the AI percentages when compared to Control at the end of the 24 h treatment with RUT 1, 10, and 50 µM (Figure 5). Despite the similar trend, at the highest concentrations tested the registered AI values were higher in SK-MEL-28 cells (10 µM–56%; 50 µM–84.19%) as compared to RPMI-7951 cells (10 µM–48.35%; 50 µM–75.25%). 

### 3.4. Senescence Detection

**Figure 6:**
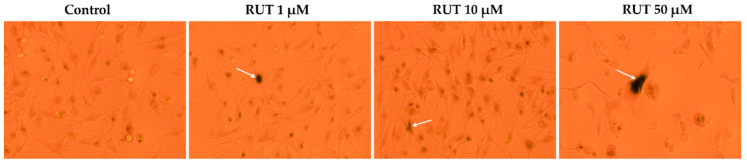
Pictures (10× magnification) of X-Gal staining of senescent SK-MEL-28 cells after a 24 h treatment with RUT (1, 10, and 50 µM). The white arrows indicate cells undergoing senescence.

The ability of RUT to induce senescence in melanoma cells has been assessed by the X-Gal staining. As the most significant viability results were noticed in SK-MEL-28 cells, this experiment was performed using this particular cell line. In comparison to Control where several senescent cells were detected as well, RUT induced an enhancement in the senescence signal (the dark color reporting β-galactosidase expression). The uppermost results were obtained at the highest concentration tested—50 µM (Figure 6).

## 4. Discussion

The present study was conducted to gather novel data regarding the effects of RUT as a potential anti-melanoma agent, data that are rather scarce at present. The main findings in this direction are as follows: (i) a dose-dependent cytotoxic effect at micromolar concentrations (1–50 µM—Figure 1) defined by apoptotic-specific features (cellular shape and nuclear alterations—Figure 2, Figure 3 and Figure 4) and (ii) a concentration-dependent senescence-inducing activity (Figure 6).

RUT, the flavonoid glycoside was also known as rutoside, displays a plethora of biological activities, including antioxidant, vasoprotective, neuroprotective, anticonvulsant, antidepressant, analgesic and antinociceptive, antidiabetic, anti-hypercholesterolemic, anticoagulant, antiulcer, antiosteoporotic, anticancer, and many other pharmacological effects that were comprehensibly described in an excellent review [26]. In recent years, a great interest was attributed to elucidate the molecular mechanisms underlying the anticancer effect of RUT [27,28], but this process is far from being elucidated. Significant steps were made in this direction and several anticancer mechanisms of action were discovered, such as (i) activation of cancer cells apoptosis in a caspase-independent pathway, (ii) induction of cell cycle arrest; (iii) modulation of different signaling pathways (e.g., Wnt, JAK-STAT, EGFR, AP-1, NF-κB, Akt); (iv) inhibition of cancer cells’ migration; (v) antiangiogenic effect, etc. These findings are based on studies conducted both in vitro and in vivo on different models of cancer such as breast cancer, lung cancer, colon cancer, hepatic cancer, neuroblastoma, leukemia, and ovarian cancer [26,27,28].

With regards to RUT’s anti-melanoma mechanism of action, the information is rather insufficient at present. A recent in vitro study reported that RUT (5–100 µg/mL) in combination with photodynamic therapy and methylene blue induced apoptosis and cell cycle arrest via ROS generation in A375 human melanoma [29]. Other data regarding the impact of RUT in melanoma were achieved from two in vivo studies that showed an antimetastatic effect of RUT by decreasing the number of metastatic nodules in a mouse melanoma model induced by injection of B16F10 [30] and an inhibition of melanin formation, but an increase in growth rate and tumor weight in C57BL/6 mice inoculated with B16 melanoma cells [31].

In the light of the data stated above and based on our previous background on the topic [15,23], this study was focused on identifying novel insights regarding the anti-melanoma effect of RUT. Our results showed that a 24 h treatment with different concentrations of RUT (1, 5, 10, 25, and 50 µM) induced a dose-dependent cytotoxic effect in both types of human melanoma cells tested—RPMI-7951 and SK-MEL-28 and morphological and nuclear alterations (nuclear fragmentation, membrane blebbing, chromatin condensation, and apoptotic bodies), characteristic signs of apoptosis (Figure 1, Figure 2, Figure 3 and Figure 4). These results are similar to the ones described in a study conducted on A375 melanoma cells [23]. Previous publications also reported the in vitro antitumor and proapoptotic properties of RUT. One such example is the paper published by Danciu et al., revealing the potency of RUT (100 µM) in decreasing the viability of B164A5 murine malignant melanoma cells after 72 h of treatment [16]. An additional study by Khan et al. reported the ability of RUT to reduce the cell viability, induce nuclear condensation, cell cycle arrest at G0/G1 phase, and apoptosis via caspase-3 activation in HPV-C33A cervical cancer cells [32]. A proapoptotic effect of RUT in A549 human lung cancer cells was also described [33]. RUT demonstrated anticancer effects also against neuroblastoma, leukemia, breast, colon, hepatic, pancreatic, and ovarian cancer cells [27].

Despite the similarities in terms of viability trend and percentages observed in both cell lines, SK-MEL-28 cells (IC_50_ = 47.44 ± 2.41) showed an augmented sensitivity to RUT as compared to RPMI-7951 cells (IC_50_ = 64.49 ± 13.27). This different response to RUT treatment could be explained by the distinct features of the two human melanoma cell lines analyzed. RPMI-7951 cells are melanotic epithelial-like cells with adherent properties that present melanosome granules and possess the capacity to synthesize melanin in vitro and to transport it from the mature melanosomes to keratinocytes [34] and to develop pigmented tumors in vivo. Moreover, according to the manufacturer (ATCC) characterization datasheet, RPMI-7951 cells harbor BRAF, PTEN, and TP53 mutant genes. By contrast, SK-MEL-28 cells are amelanotic cells presenting a melanocyte-like phenotype with polygonal morphology, capable to form large round cell type melanoma in vivo. These cells express as mutant genes BRAF, TP53, and CDK4 [35] and cannot produce melanin in vitro as shown in our previous study [36]. Recently, it has been highlighted that melanin plays a key role in the protection of pigmented cells against the injuries induced by chemical toxicants [37]. Melanin is a negatively charged biopolymer that colors various human structures such as skin, hair, and eyes [38]. The main biological function of melanin is to exert a cytoprotective effect against noxious UV radiations by serving as a physical barrier and absorbent filter that reduces their penetration through the epidermis [39], as well as against oxidative stress damage by scavenging free radicals [40]. Hence, melanin can bind small molecules, leading to their retention in the pigmented tissues [40] followed by a slow release of the accumulated toxins [37]. In particular, melanin interacts by hydrogen and π−π bonding with small aromatic molecules [40] such as RUT. 

Besides the visualization of nuclear alterations induced by RUT treatment in both cell lines—RPMI-7951 and SK-MEL-28 (Figure 3 and Figure 4), the Hoechst 33342 staining assay was used to calculate the apoptotic index value (AI—Figure 5). Our results indicated a similar trend as to that observed for the viability results: a concentration-dependent elevation in the apoptotic index values (Figure 5). The highest percentages were recorded following the 24 h treatment with RUT 50 µM in both cell lines: RPMI-7951—75.25%; SK-MEL-28—84.19%. Regarding the safety profile of RUT, one of our latest publications reveals that RUT induces no cytotoxicity in normal keratinocytes—the predominant cellular component in human skin [41], even at high concentrations (50 and 75 µM) [23]. 

An interesting and novel finding in the present study is represented by the senescence-inducing activity exerted by RUT after a 24 h treatment in SK-MEL-28 cells (Figure 6), results that were not reported before to the best of our knowledge. 

Cellular senescence is described as a very complex and heterogeneous process due to its involvement in multiple physiological and pathological conditions (e.g., aging and cancer) being characterized by several specific features as cell-cycle abolition, macromolecular damage, impaired metabolism, and a secretory phenotype [42,43]. At present, cellular senescence is considered (i) a tumor-suppressive process by preventing malignant transformation; (ii) an effector mechanism of current chemotherapy, and (iii) an active process in preventing disease recurrence following cancer treatment [43]. Therefore, an X-Gal staining protocol has been applied after the 24 h stimulation of SK-MEL-28 cells with RUT. The most prominent results were acquired at the highest concentration of 50 µM (Figure 6) when specific senescence features such as increased cell size, and elevated senescent-associated beta-galactosidase (SA-β-gal) expression [44] highlighted by a dark coloration within the cell cytoplasm were detected. Our results are endorsed by previous studies that reported the capacity of several natural bioactive compounds (e.g., curcumin, quercetin) to force cancer cells to undergo senescence in vitro [45,46,47]. Multiple mechanistic pathways have been attributed to the pro-senescence effect of natural polyphenols in cancer cells, including modulation of tumor suppressor or oncogene gene expression, DDR (DNA damage response) activation, ROS generation, promotion of endoplasmic reticulum (ER) stress, and regulation of epigenetics [48]. According to our results, RUT treatment-induced senescence in SK-MEL-28 melanoma cells by augmenting the expression of beta-galactosidase (SA-β-gal), but the underlying mechanism is far more complex. Based on the data from the literature regarding the signaling pathways modulated by RUT in exerting its anticancer effect (mitogen-activated protein kinase (MAPK), PI3K/Akt, Wnt/β-catenin cascade, Janus kinase, Ras/Raf, TGF-β2/Smad2/3Akt/PTEN, epidermal growth factor (EGF) pathway, p53, etc.) [27] and the data regarding the mechanisms involved in cellular senescence of cancer cells [42,43], we could assume that RUT acts by targeting various pathways as p53 pathway, PI3K/Akt and others, but these assumptions need further studies for attestation. 

## 5. Conclusions

Our findings revealed that RUT possesses a dose-dependent cytotoxic activity at micromolar concentrations against human melanoma cells. The cytotoxic effect was associated with a reduced cell viability rate, changes in cellular morphology, apoptotic-like nuclear alterations, and reduced confluence. Moreover, RUT enhanced the senescence within SK-MEL-28 cells at the highest concentrations (10 and 50 µM). Further studies are necessary to confirm and elucidate the mechanisms underlying the antiproliferative and pro-senescent properties of RUT in melanoma cells. 

## Data Availability

The data presented in this study are available on request from the corresponding author.
